# Hirsutanone Isolated from the Bark of *Alnus japonica* Attenuates Melanogenesis via Dual Inhibition of Tyrosinase Activity and Expression of Melanogenic Proteins

**DOI:** 10.3390/plants11141875

**Published:** 2022-07-19

**Authors:** Takuhiro Uto, Nguyen Huu Tung, Yukihiro Shoyama

**Affiliations:** 1 Department of Pharmacognosy, Faculty of Pharmaceutical Sciences, Nagasaki International University, 2825-7 Huis Ten Bosch, Sasebo, Nagasaki 859-3298, Japan; shoyama@niu.ac.jp; 2 Faculty of Pharmacy, Phenikaa University, Hanoi 100000, Vietnam; tung.nguyenhuu@phenikaa-uni.edu.vn

**Keywords:** *Alnus japonica*, hirsutanone, diarylheptanoid, anti-melanogenesis, tyrosinase, B16-F1 murine melanoma, normal human epidermal melanocytes, melanogenic enzymes, melanogenic enzymes

## Abstract

Hirsutanone (Hir) and oregonin (Ore) are diarylheptanoids isolated from the bark of *Alnus japonica*. In this study, we investigated the anti-melanogenic activity of Hir and Ore in B16-F1 murine melanoma and normal human epidermal melanocytes (HEMn-DP) and elucidated the mechanisms of action. In B16-F1 cells, Hir and Ore suppressed melanin synthesis induced by α-melanocyte-stimulating hormone (α-MSH) without cytotoxicity. The inhibitory effect of Hir on melanin synthesis was much stronger than that of Ore. In addition, Hir reduced melanin content in HEMn-DP cells. As tyrosinase is a key enzyme in melanin synthesis, the effect of Hir on tyrosinase activity was assessed. The results demonstrated that Hir partially decreased tyrosinase activity and intracellular tyrosinase activity. Moreover, Hir suppressed the protein expression of melanogenic enzymes, including tyrosinase, tyrosinase-related protein (TRP)-1, and TRP-2, leading to reduced melanin biosynthesis. Hir also led to the suppression of cAMP response element-binding protein (CREB) phosphorylation and microphthalmia-associated transcription factor (MITF) expression, which control the expression of melanogenic enzymes. These results suggest that Hir suppressed melanin synthesis by dual inhibition of tyrosinase activity and the CREB/MITF pathway leading to the expression of melanogenic enzymes and may be a potent cosmetic and therapeutic agent for hyperpigmentation disorders.

## 1. Introduction

In mammals, skin color is determined by the amount of melanin on the skin surface [1,2]. Melanin plays an important role in protecting the skin from the harmful effects of ultraviolet radiation and oxidative stress caused by various environmental pollutants. However, excessive production of melanin causes various hyperpigmentation disorders, such as freckles, solar lentigo (age spots), melasma, and cancer [3,4]. These conditions can lead to a deterioration in quality of life and are clinical and cosmetic concerns worldwide. Therefore, the development of melanin production inhibitors is important for controlling hyperpigmentation, and considerable efforts have led to the discovery of many melanin inhibitors, such as hydroquinone [5], kojic acid [6,7], and arbutin [8,9].

Melanogenesis is a biosynthetic pathway that produces melanin pigments in the melanocytes. Melanogenesis is regulated by three essential melanogenic enzymes: tyrosinase, tyrosinase related protein (TRP)-1, and TRP-2 [10]. Tyrosinase is a rate-limiting enzyme that catalyzes the first two steps of melanin biosynthesis: hydroxylation of tyrosine to 3,4-dihydroxyphenylalanine (DOPA) and oxidation of DOPA to dopaquinone [11]. Dopaquinone is spontaneously converted to dopachrome. TRP-2, which functions as a dopachrome tautomerase, catalyzes the conversion of dopachrome to 5,6-dihydroxyindole-2-carboxylic acid (DHICA). Subsequently, TRP-1 oxidizes DHICA to produce carboxylate indole-quinone [12]. These enzymes upregulate and activate melanin biosynthesis. The microphthalmia-associated transcription factor (MITF), a major transcription factor in melanocytes, binds to an M-box motif in the promoter region of three essential melanogenic genes and upregulates their transcription [13]. Various pathways can regulate melanogenic gene expression. Phosphorylated cAMP response element-binding protein (CREB) is one of the major stimulatory signals that activate MITF transcriptional activity [14]. Phosphorylated CREB binds to the cAMP response element (CRE) present in the MITF promoter [14]. Finally, the increase in MITF levels leads to the upregulation of tyrosinase, TRP-1, and TRP-2, thereby promoting melanin biosynthesis. Therefore, inhibition of this signaling pathway may improve hyperpigmentation disorders.

**Figure 1:**
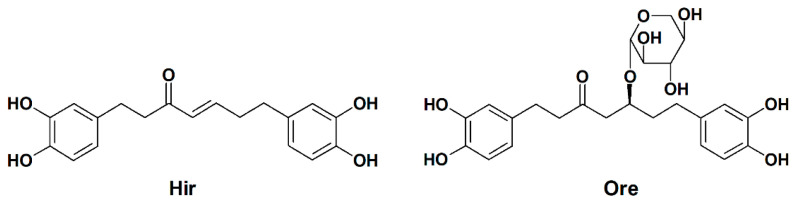
Chemical structures of Hir and Ore.

*Alnus japonica* Steud (Betulaceae, Japanese name: Hannoki, English name: East Asian alder) is a broad-leaved tree widely distributed in Japan, Korea, and North China, and its bark is used in traditional Asian medicine for fever, hemorrhage, diarrhea, alcoholism, various skin infections, and inflammation [15]. A previous phytochemical analysis of *A. japonica* revealed the presence of diarylheptanoids, triterpenoids, and flavonoids [16,17,18,19]. Among them, hirsutanone (Hir) and oregonin (Ore) (Figure 1) are the major diarylheptanoids isolated from *A. japonica* bark and have been reported to possess significant bioactivities. Our previous studies revealed the antioxidative and anti-influenza properties of diarylheptanoids, including Hir and Ore [20,21]. We reported that Hir and Ore potently inhibit the growth of *Trypanosoma brucei* in the bloodstream [22]. Furthermore, Ore inhibits ischemia-reperfusion-induced mesentery oxidative stress by inhibiting nicotinamide adenine dinucleotide phosphate (NADPH) oxidase activation [23]. Jeong et al. reported that Hir suppresses early T-cell activation, thereby inhibiting the degranulation of mast cells, making it a potential candidate for treating atopic dermatitis [24]. Park et al. demonstrated that Hir exhibits strong papain-like protease inhibitory activity in suppressing the replication of severe acute respiratory syndrome coronavirus (SARS-CoV) [25]. Furthermore, the anticancer activities of Hir and Ore have been reported in several cancer cell lines, including colon cancer, prostate cancer, ovarian cancer, and leukemia [18,26,27,28]. However, to date, no studies have focused on the effects of Hir or Ore on melanin biosynthesis.

In a preliminary study, we screened approximately 130 crude drugs used in Kampo formulas and 250 natural compounds to identify regulators of melanogenesis. Based on the screening results, we reported several stimulators of melanin biosynthesis, such as liquiritin and liquiritigenin (flavonoids in licorice root) [29], (+)-magnolin (lignan in the flower buds of *Magnolia biondii*) [30], silibinin (flavonolignan in milk thistle) [31], and eugenol (phenylpropanoid in clove buds) [32]. The screening results also indicated that Hir and Ore notably suppressed melanin synthesis in melanoma cells. Therefore, in this study, we aimed to evaluate the effects of Hir and Ore on melanin biosynthesis in murine melanoma B16-F1 cells and HEMn-DP cells. In addition, we investigated enzyme activity and protein expression involved in melanogenesis. Our results suggest that Hir is an effective anti-melanogenic agent. To the best of our knowledge, this is the first study to describe the effects of diarylheptanoids from *A. japonica* bark on melanogenesis inhibition.

## 2. Results and Discussion

### 2.1. Effects of Hir and Ore on Melanin Biosynthesis and Cell Viability in B16-F1 Cells

**Figure 2:**
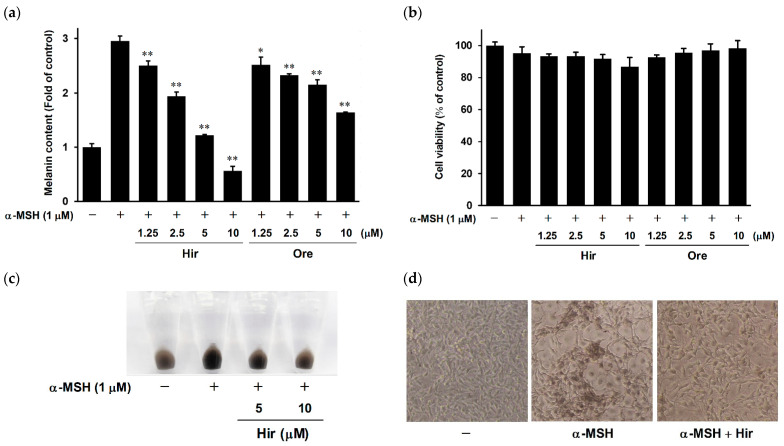
Effects of Hir and Ore on melanin synthesis and cell viability in B16-F1 cells. The cells were treated with Hir or Ore at the indicated concentrations for 1 h, then stimulated with α-MSH for 72 h. (**a**) Melanin content was determined as described in Materials and Methods. Values are the mean ± SD of three independent experiments. * *p* < 0.05 and ** *p* < 0.01 versus α-MSH. (**b**) Cell viability was determined as described in Materials and Methods. There were no significant differences between the α-MSH and experimental groups (*p* > 0.05). (**c**) Photograph of cell pellets. (**d**) Microscopic views of cells.

First, we examined the inhibitory effects of Hir and Ore on α-melanocyte-stimulating hormone (α-MSH)-induced melanin biosynthesis in murine melanoma B16-F1 cells. Cells were treated with Hir or Ore at several doses (1.25, 2.5, 5, or 10 μM) for 1 h, followed by α-MSH treatment. As shown in Figure 2a, upon exposure to α-MSH alone indicated as + (second bar from left), the melanin content increased by approximately three-fold compared to the control group indicated as − (leftmost bar). Hir and Ore significantly decreased the melanin content in a dose-dependent manner. The IC_50_ values of Hir and Ore were 3.87 µM and 16.71 µM, respectively. To exclude the possibility that the inhibitory effects of Hir and Ore might be caused by inhibition of cell growth, we determined the cytotoxicity of Hir and Ore in B16-F1 cells using a 3-(4,5-dimethylthiazol-2-yl)-2,5-diphenyltetrazolium bromide (MTT) assay. As shown in Figure 2b, Hir and Ore did not affect cell viability at concentrations as high as 10 µM, indicating that their effects were not attributable to the inhibition of cell growth. Figure 2c,d show cell pellets and microscopic views of B16-F1 cells, respectively. The pigmentation of cells treated with Hir was considerably lower than that of cells treated with α-MSH alone. These results indicate that Hir and Ore reduced melanin synthesis in B16-F1 cells without inhibiting cell growth, and the inhibitory potency of Hir was higher than that of Ore.

### 2.2. Effect of Hir on Melanin Biosynthesis and Cell Viability in Human Epidermal Melanocytes

**Figure 3:**
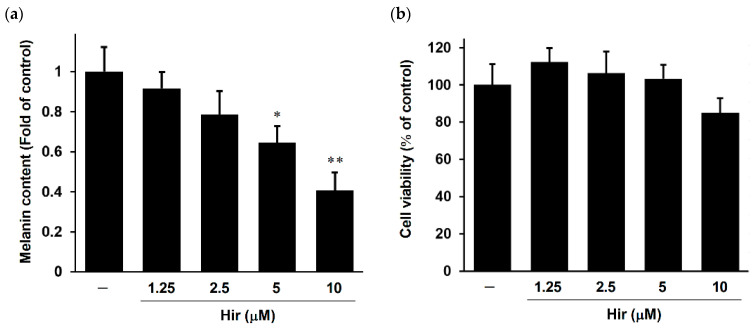
Effect of Hir on melanin biosynthesis and cell viability in HEMn-DP cells. The cells were treated with Hir at the indicated concentrations for 72 h. (**a**) Melanin content was determined as described in Materials and Methods. Values are the mean ± SD of three independent experiments. * *p* < 0.05 and ** *p* < 0.01 versus control. (**b**) Cell viability was determined as described in Materials and Methods. There were no significant differences between the control and experimental groups (*p* > 0.05).

To examine the effect of Hir on melanin biosynthesis in human melanocytes as an in vitro experimental model system, we measured melanin content and cell viability using normal human dark-pigmented melanocytes (HEMn-DP). As shown in Figure 3a, Hir decreased melanin content in a dose-dependent manner in HEMn-DP cells and in a manner similar to that in B16-F1 cells. Furthermore, Hir showed no cytotoxic effects on cell viability over the tested concentration range (Figure 3b). These results confirmed the anti-melanogenic activity of Hir in normal human melanocytes and melanoma cells.

### 2.3. Effect of Hir on Tyrosinase Activity

**Figure 4:**
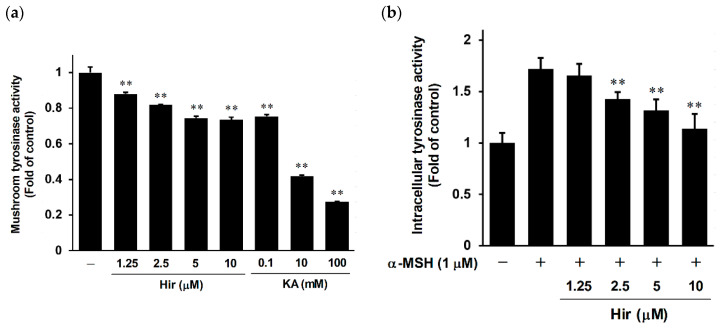
Effect of Hir on tyrosinase activity. (**a**) Mushroom tyrosinase activity was determined as described in Materials and Methods. Kojic acid (KA) was used as a positive control. Values are the mean ± SD of three independent experiments. ** *p* < 0.01 versus control. (**b**) B16-F1 cells were treated with Hir at the indicated concentrations for 72 h, and intracellular tyrosinase activity was determined as described in Materials and Methods. Values are the mean ± SD of three independent experiments. ** *p* < 0.01 versus α-MSH.

Tyrosinase is a key enzyme that controls melanogenesis [10]. The suppression of melanin biosynthesis can be achieved by inhibiting tyrosinase activity or reducing the protein expression of melanogenic enzymes, including tyrosinase [5,6,7,8,9,10]. Therefore, we examined the direct inhibitory effect of Hir on tyrosinase activity using a cell-free mushroom tyrosinase system. Kojic acid (KA) was used as a positive control to directly inhibit tyrosinase activity [33]. As shown in Figure 4a, Hir had an inhibitory effect on the oxidation activity of mushroom tyrosinase in a dose-dependent manner. Furthermore, in the cell-based tyrosinase assay, inhibition of intracellular tyrosinase activity occurred in a dose-dependent manner in B16-F1 cells (Figure 4b). These results suggest that Hir has anti-melanogenic activity via, at least in part, direct inhibition of tyrosinase activity. However, the inhibitory potency of Hir on cell-free mushroom tyrosinase seems weak since Hir at 10 μM reduced activity by 0.73-fold, compared to KA inhibiting mushroom tyrosinase activity by 0.27-fold. Nonetheless, these results implied that Hir attenuated the protein expression of melanogenic enzymes while simultaneously inhibiting tyrosinase activity directly.

### 2.4. Effects of Hir on Expression of Tyrosinase, TRP-1, and TRP-2 in B16-F1 Cells

**Figure 5:**
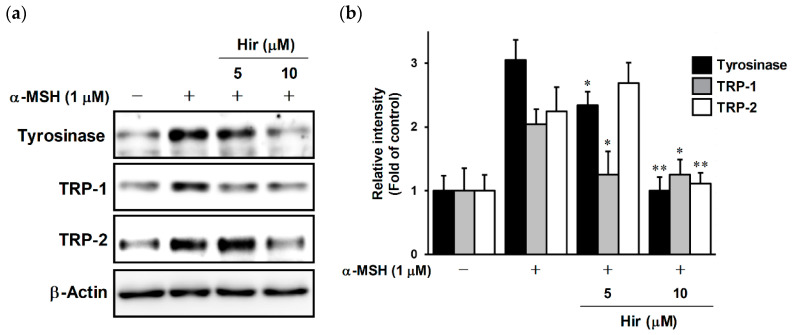
Effect of Hir on the protein levels of tyrosinase, TRP-1, and TRP-2 in B16-F1 cells. (**a**) The cells were treated with Hir at the indicated concentrations for 1 h, then stimulated with α-MSH for 48 h. Protein expression was determined by Western blotting as described in Materials and Methods. (**b**) Band intensities were quantified using ImageJ software and normalized to the band intensities of β-action as an internal control. The data shown are representative of 3–4 independent experiments. * *p* < 0.05 and ** *p* < 0.01 versus α-MSH.

We conducted a Western blot experiment to evaluate changes in the protein levels of tyrosinase family members, including tyrosinase, TRP-1, and TRP-2 (Figure 5a). Treatment with α-MSH increased the levels of tyrosinase, TRP-1, and TRP-2, whereas Hir inhibited the expression of these proteins. The relative intensity of protein bands is shown after normalization with β-actin (Figure 5b). These results suggest that Hir inhibited melanin biosynthesis by decreasing the protein expression of tyrosinase, TRP-1, and TRP-2, with direct inhibition of tyrosinase activity.

### 2.5. Effects of Hir on MITF Expression and CREB Phosphorylation in B16-F1 Cells

**Figure 6:**
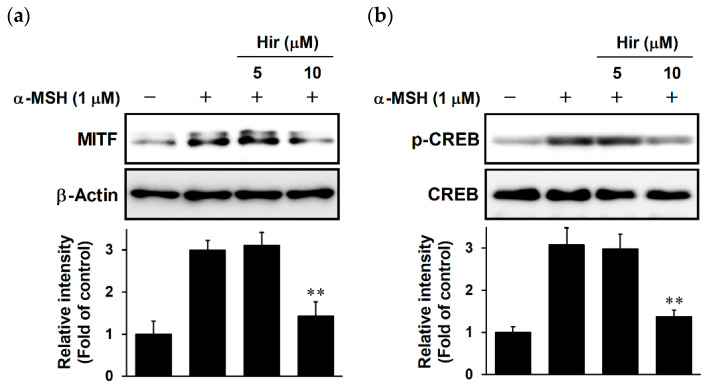
Effect of Hir on MITF expression and CREB phosphorylation in B16-F1 cells. The cells were treated with Hir at the indicated concentrations for 1 h, then stimulated with α-MSH for 24 h (**a**) or 30 min (**b**). Protein expression was determined using Western blotting as described in Materials and Methods. Band intensities were quantified using ImageJ software and normalized to the band intensities of β-actin (**a**) or CREB (**b**) as the internal control. The data shown are representative of 3–4 independent experiments. ** *p* < 0.05 versus α-MSH.

MITF binds to melanogenic gene promoters, which increases the transcription of melanogenic enzymes, leading to increased melanin biosynthesis [13]. Therefore, we investigated the effect of Hir on MITF expression. As expected, Hir attenuated the protein levels of MITF induced by α-MSH (Figure 6a). CREB phosphorylation is a major signaling pathway that increases MITF expression at the transcriptional level upon α-MSH stimulation [14]. As shown in Figure 6b, CREB was significantly phosphorylated by α-MSH, and Hir suppressed this phosphorylation. These results suggest that the inhibition of melanogenic enzyme proteins by Hir may be associated with the suppression of CREB phosphorylation, leading to downregulation of MITF expression.

## 3. Materials and Methods

### 3.1. Materials

α-MSH was purchased from Sigma-Aldrich (St. Louis, MO, USA). Dulbecco’s Modified Eagle Medium (DMEM) was obtained from Nissui Pharmaceutical (Tokyo, Japan). Antibodies against tyrosinase, TRP-1, TRP-2, and β-actin were obtained from Santa Cruz Biotechnology (Santa Cruz, CA, USA). Antibodies against MITF, phosphorylated CREB, and CREB were obtained from Cell Signaling Technology (Danvers, MA). Fetal bovine serum (FBS) was obtained from Gibco (Gaithersburg, MD, USA). All other chemicals were purchased from Wako Pure Chemical Industries (Osaka, Japan).

### 3.2. Plant Material and Isolation of Hir and Ore

The bark of *A. japonica* was harvested from Kyushu University Forest in January 2011 by Prof. Takao Setsu. A voucher specimen was deposited in the Department of Pharmacognosy, Faculty of Pharmaceutical Sciences, Nagasaki International University. Hir and Ore were isolated as described previously [22]. The structures of Hir and Ore have been confirmed previously [22].

### 3.3. Cell Culture and Treatment

B16-F1 cells were obtained from the European Collection of Authenticated Cell Cultures (ECACC) through DS Pharma Biomedical (Osaka, Japan). B16-F1 cells were cultured in DMEM containing 10% FBS and 1% penicillin-streptomycin solution. HEMn-DP cells were obtained from Gibco and cultured in Medium 254 supplemented with 1% human melanocyte growth supplement (HMGS) and 1% penicillin-streptomycin-amphotericin B solution. Both cell lines were incubated at 37 °C in a humidified 5% CO_2_ atmosphere. For cell treatment, Hir and Ore were dissolved in dimethyl sulfoxide (DMSO). α-MSH was dissolved in water. These reagents were stored at −20 °C before use. The final concentration of DMSO in the medium did not exceed 0.2% (*v*/*v*) and the controls were treated with the same amount of DMSO as that used in the corresponding experiments. In experiments using α-MSH, cells were pretreated with or without Hir or Ore for 1 h before α-MSH treatment.

### 3.4. Measurement of Melanin Content and Microscopy

The cells were seeded in 24-well plates at a density of 2 × 10^4^ cells/well for B16-F1 and 5 × 10^4^ cells/well for HEMn-DP. After incubation for 24 h, cells were treated with each agent for 72 h. At the end of the treatment, B16-F1 cells were observed under a Nikon TS-1000 microscope (Nikon, Tokyo, Japan) and photographed using a Canon EOS Kiss X7i camera (Canon, Tokyo, Japan). To measure melanin content, the medium was removed, and the cells were dissolved in 120 μL of 1 M NaOH at 80 °C for 20 min. One hundred microliters of the solution were transferred to a 96-well plate, and the absorbance at 415 nm was measured using a microplate reader (iMark, BioRad, Tokyo, Japan). Melanin content was calculated from the absorbance ratio relative to that of the control culture. 

### 3.5. Cell Viability Assay

Cell viability was determined using the MTT assay [29,30,31,32]. Briefly, the cells were seeded in 96-well plates at a density of 0.3 × 10^4^ cells/well for B16-F1 or 0.8 × 10^4^ cells/well for HEMn-DP. After incubation for 24 h, cells were treated with each agent for 72 h. At the end of the treatment, 10 μL of MTT solution (5 mg/mL in phosphate-buffered saline (PBS)) was added to each well, and the cells were incubated for 4 h. Formazan precipitate was dissolved in 100 μL of 0.04 M HCl-isopropanol, and the absorbance at 595 nm was measured using an iMark microplate reader (Bio-Rad). Cell viability was expressed as a percentage of that of the control culture.

### 3.6. Measurement of Cell-Free Mushroom Tyrosinase Activity

Cell-free mushroom tyrosinase activity was assayed by measuring DOPA oxidase activity as described previously, with slight modifications [34]. Briefly, 80 μL of buffer (0.1 M phosphate buffer, pH 6.8), 40 µL of each agent, 40 µL of the aqueous solution of mushroom tyrosinase (92 UI/mL), and 40 µL of L-DOPA (5 mM) were placed into a 96-well plate. Kojic acid was used as a positive control. The reaction mixture was incubated at 37 °C for 10 min, and the absorbance at 490 nm was measured using an iMark microplate reader (Bio-Rad). Mushroom tyrosinase activity was expressed as a ratio of the control value.

### 3.7. Assay of Intracellular Tyrosinase Activity 

Intracellular tyrosinase activity was determined by measuring the rate of dopachrome production from L-DOPA, as described previously [29,30,31,32]. B16-F1 cells were seeded in 6 cm dishes at a density of 3 × 10^5^ cells/dish. After incubation for 24 h, cells were treated with each agent for 72 h. At the end of treatment, the cells were washed twice with ice-cold PBS and lysed in 600 μL of 0.1 M sodium phosphate buffer (pH 6.8) containing 1% Triton X-100 and a proteinase inhibitor cocktail. The lysate was clarified by centrifugation at 12,000× *g* for 15 min at 4 °C, and the supernatant was collected. After measuring the protein concentration using a dye-binding protein assay kit (Bio-Rad), 90 μL of equal amounts of protein lysate and 10 µL of 5 mM L-DOPA were added to each well of a 96-well plate. During incubation at 37 °C in the dark, the generated dopachrome was monitored using an iMark microplate reader (Bio-Rad) at an absorbance of 415 nm. Tyrosinase activity was expressed as a ratio of the control values.

### 3.8. Western Blot Analysis 

B16-F1 cells were seeded in 6-cm dishes at a density of 3 × 10^5^ cells/dish. After incubation for 24 h, the cells were treated with Hir, followed by treatment with α-MSH. At the end of the incubation period, cells were washed twice with ice-cold PBS and lysed in RIPA lysis buffer containing protease and phosphatase inhibitors. The protein concentration was measured using a dye-binding protein assay kit (Bio-Rad). Equal amounts of the protein lysate were mixed with sodium dodecyl sulfate (SDS) sample buffer and boiled for 10 min. Proteins were separated by SDS-polyacrylamide gel electrophoresis and transferred to polyvinylidene fluoride membranes. The membranes were blocked with 5% skim milk in a Tris-buffered saline with 0.1% Tween 20 (TBS-T) buffer and incubated with specific primary antibodies at 4 °C overnight. After washing with TBS-T, membranes were incubated with horseradish peroxidase-conjugated secondary antibodies for 1 h. After washing with TBS-T, the proteins were detected using ECL Prime Western blotting detection reagent and analyzed using the Image Quant LAS-4000 mini system (GE Healthcare Bioscience, Piscataway, NJ, USA). Band intensities were quantified using ImageJ software (version 1.53k; National Institutes of Health) and normalized to the corresponding loading control.

### 3.9. Statistical Analysis

All the data were obtained from at least three independent treatment repetitions. The results are expressed as the mean ± SD under each condition. Data were analyzed by one-way ANOVA followed by Tukey’s test using GraphPad Prism 6 software (San Diego, CA, USA), and statistical significance was set at *p* < 0.05.

## 4. Conclusions

Hir and Ore inhibited melanin biosynthesis in α-MSH-stimulated B16-F1 cells without inhibiting cell growth, and the inhibitory potency of Hir was higher than that of Ore. Similarly, Hir reduced melanin synthesis in normal human melanocytes (HEMn-DP) cells. To further confirm the anti-melanogenic activity of Hir, animal studies and clinical trials are needed in future studies. Further analysis demonstrated that Hir significantly suppressed cell-free tyrosinase activity and intracellular tyrosinase activity in B16-F1 cells. In addition, the protein expression of tyrosinase, TRP-1, and TRP-2 was inhibited by Hir treatment. Hir also suppressed MITF expression and CREB phosphorylation. Taken together, Hir exerts anti-melanogenic activity through dual inhibition of tyrosinase activity and the CREB/MITF pathway leading to the reduced expression of melanogenic enzymes. This study is the first to demonstrate the anti-melanogenic activity of Hir and its underlying molecular mechanism. Our results suggest that Hir could be developed as a potent cosmetic and therapeutic agent for hyperpigmentation disorders.

## Data Availability

Not applicable.
